# Factorial validity of the Twi versions of five measures of mental health and well-being in Ghana

**DOI:** 10.1371/journal.pone.0236707

**Published:** 2020-08-11

**Authors:** Richard Appiah, Lusilda Schutte, Angelina Wilson Fadiji, Marié P. Wissing, Amanda Cromhout

**Affiliations:** 1 Africa Unit for Transdisciplinary Health Research, Faculty of Health Sciences, North-West University, Potchefstroom, South Africa; 2 College of Health Sciences, University of Ghana, Accra, Ghana; 3 Inclusive Economic Development, Human Sciences Research Council, Cape Town, South Africa; Universidad Autonoma de Madrid, SPAIN

## Abstract

**Background:**

Mental health is considered an integral part of human health. Reliable and valid measurement instruments are needed to assess various facets of mental health in the native language of the people involved. This paper reports on five studies examining evidence for the factorial validity of the Twi versions of five mental health and well-being measurement instruments: Affectometer-2 (AFM-2); Automatic Thought Questionnaire–Positive (ATQ-P); Generalized Self-Efficacy Scale (GSEs); Patient Health Questionnaire-9 (PHQ-9); and Satisfaction with Life Scale (SWLS) in a rural Ghanaian adult sample.

**Method:**

Measures were translated and evaluated using a research-committee approach, pilot-tested, and administered to adults (*N* = 444) randomly selected from four rural poor communities in Ghana. We applied confirmatory factor analysis (CFA), bifactor CFA, exploratory structural equation modeling (ESEM), and bifactor ESEM to the AFM-2, ATQ-P, and the PHQ-9, and CFA to the GSEs and the SWLS. The omega coefficient of composite reliability was computed for each measure.

**Results:**

A two-factor bifactor ESEM model displayed superior model fit for the AFM-2. The total scale and the Negative Affect subscale, but not the Positive Affect subscale, attained sufficient reliability. Two models (a four-factor 22-item bifactor ESEM model and a 5-factor 22-item ESEM model) fitted the data best for the ATQ-P. The bifactor ESEM model displayed a high reliability value for the total scale and satisfactory reliability values for three of its four subscales. For the GSEs, a one-factor CFA model (residuals of items 4 and 5 correlated) demonstrated superior model fit with a high reliability score for the total scale. A two-factor ESEM model outperformed all other models fitted for the PHQ-9, with moderate and satisfactory reliability scores for the subscales. A one-factor CFA model (residuals of item 4 and 5 correlated) demonstrated superior model fit for the SWLS, with a satisfactory reliability value for the total scale.

**Conclusions:**

Findings established evidence for the factorial validity of the Twi versions of all five measures, with the global scores, but not all subscale scores, demonstrating satisfactory reliability. These validated measurement instruments can be used to assess mental health and well-being in the research and practice contexts of the current sample.

## Introduction

There is a growing global interest in strengths-based research that focuses on the promotion of positive human functioning [1, 2]. Recent scholarly efforts have expanded from the traditional foci of assessing and treating psychopathology to also evaluating and promoting people’s strengths and mental health [3, 4]. This has led to a rapid increase in the development of several measurement instruments that assess the strengths, capacities, and mental health of people in different contexts. A comprehensive assessment of positive mental health involves a consideration of both hedonic and eudaimonic aspects of well-being. Generally, hedonia emphasizes pleasure, subjective happiness, and avoidance of negative affect [3, 5]. Eudaimonia, on the other hand, focuses on aspects such as purpose in life, self-actualization, and personal growth [6, 7]. There is also a growing recognition of the complex interactions between positive aspects of human functioning and negative experiences that influence thinking patterns, feelings, and behavior of people [8, 9]. Consequently, a complete assessment of mental health is complemented by the evaluation of negative experiences and emotional states, such as depression and negative affect [9].

Recent developments in the field of positive psychology have led to renewed research interest in exploring evidence for the validity of measurement instruments aimed at measuring constructs such as mental well-being, satisfaction with life, and self-efficacy across populations and contexts [4, 10]. The majority of these measurement instruments in use in most African settings were developed and validated from a Western perspective, assumed individualistic cultural orientation and values, and were largely validated with unrepresentative Western samples [11]. Owing to vast cultural differences that exist between people from different social structures and value orientations, for instance, between Western and African societies [11, 12], it is important that the evidence for the validity of measurement instruments that were developed and normed in one context (e.g., Western) are established before they are administered in other (e.g., African) contexts. Evidence suggests that the conceptualization, interpretation, and expression of well-being differ between individualistic (e.g., Euro-American) and collectivistic (e.g., African and Asian) cultures [13].

Although several mental health and well-being measurement instruments are used for research and clinical practice in Ghana, very few, such as the General Health Questionnaire-12 [14], Perceived Social Support from Family and Friends Scale [15], and the Multidimensional Scale of Perceived Social Support [16] have been validated in their original English versions in the Ghanaian context. Furthermore, given that the meaning or manifestation of constructs may differ across cultural groups, it is important that measurement instruments are translated and the evidence for their validity established in the languages and cultural contexts of the target population. Empirically validated versions of mental health and well-being measurement instruments in the native languages can strengthen the drive towards ensuring valid, reliable, and culturally-appropriate assessment of various constructs of mental health. This would also provide opportunities for researchers to empirically evaluate mental health intervention programs in the Ghanaian context. Furthermore, in-depth information on the nature and prevalence of mental health and well-being for a particular group can serve as a useful resource for designing context-appropriate and cost-effective interventions for the people involved.

### The present study

Although the Affectometer-2 (AFM-2), Automatic Thought Questionnaire–Positive (ATQ-P), Generalized Self-Efficacy scale (GSEs), Patient Health Questionnaire–9 (PHQ-9), and Satisfaction with Life Scale (SWLS) are often used to assess mental health and well-being in research and practice in Ghana, their psychometric properties have not been investigated in any Ghanaian population, at present. This paper reports on five studies examining the evidence for the factorial validity of the Twi versions of five mental health and well-being measures in a sample of adults drawn from four rural poor communities in the middle belt of Ghana. We first present a general description of the methodology implemented in all five studies. This is followed by a sequential presentation of each validated measurement instrument, together with their results and discussion of findings.

## General method

### Design and participants

A once-off cross-sectional survey design was implemented to collect data for the five studies. Participants (*N* = 444) were Twi-speaking adults drawn from four rural poor communities from the Sunyani West District (SWD) of the Brong Ahafo region of Ghana. Measures were interviewer-administered in the native language (Twi) of participants.

### Study setting

The SWD is one of 27 districts in the Brong Ahafo region with a population of 85,272 and a total number of 10,715 households [17]. Four communities were randomly selected from a list of 18 communities categorized as extremely poor (with an average of 150 households and an income per head below 50% of the poverty line of US$1.90 a day) or classified as ultra-poor (with US$ 1.25 or less a day) [18–20]. Most of these communities have poor road networks or footpaths connecting them and have only basic (primary) schools. Although most communities are connected to the national grid, only about half of the households are connected to electricity. Residents are mainly peasant farmers and traders of farm produce who also share similar socioeconomic characteristics [17, 18].

### Measures

Five mental health and well-being measures commonly used in Ghana, including the Affectometer-2 (AFM-2), Automatic Thoughts Questionnaire-Positive (ATQ-P), Generalized Self-Efficacy Scale (GSEs), Patient Health Questionnaire-9 (PHQ-9), and Satisfaction with Life Scale (SWLS) were selected to examine the evidence for their factorial validity in this study. Presently, there is a general lack of research that investigates the evidence for the validity of the Twi version of mental health and well-being measurement instruments in Ghana, particularly in a rural poor context. The selected measurement instruments have shown promise in previous African studies [21–23, 47, 49], are all relatively short, and together assess facets relevant to the evaluation of mental health and well-being in the African context. The selection of these measures was also supported by the growing research evidence that suggests that the complete evaluation of mental health and well-being should also be complemented by the assessment of negative experiences and emotional states [9], considering the complex interactions between positive aspects of human functioning and negative experiences [8, 9] that, together, influence thinking patterns, feelings, and behavior of people.

#### Sociodemographic data

We collected sociodemographic information including gender, age, education, marital status, and income.

### Procedure

#### Preparation of scales

We followed the research-committee approach [24, 25] to translate the measures from English to Twi. First, the measures were translated into Twi by a translator and back-translated into English by another. The English versions (i.e., the original English version and the Twi-English back-translated version) were then compared and evaluated by a panel of academics including a linguistic (forward translator), an English professor also fluent in Twi (back-translator), the first author (who is a clinical psychologist and a native Twi speaker), and an independent member (who is fluent in Twi and English). To ensure that the translated versions were comprehensible and culturally-sensitive, we conducted a pilot study with a small group of 38 participants drawn from the target population. Participants rated the instructions, items, and the response format as “clear” or “unclear”, and indicated their understanding of the instructions, items, and response formats [26]. Participants who rated instructions, items, or the response format as “unclear” were asked to suggest a revision. Overall, the evaluation of the Twi-English back-translated versions showed two differences in meaning in the wording of the ATQ-P and one difference in meaning in the wording of the SWLS. In all instances, the research committee evaluated the differences, reached consensus, and applied the necessary revisions. See S1 File for the final Twi versions of the scales.

#### Fieldwork and data gathering

Measures were interviewer-administered by four trained psychology graduates fluent in Twi. Prior to the interviews, copies of ethical approval documents and permission letters were presented to the chiefs and community leaders of the communities involved for permission to conduct the study. With assistance from community leaders, the research team recruited an individual who acted as an independent mediator to introduce the researchers and the study to households a week before data gathering. Written informed consent was obtained from individuals who met the inclusion criteria and agreed to participate. Interviews were conducted in the homes of participants at a time that were convenient to them and at a place that ensured privacy. Data were collected electronically with the SurveyCTO software within a period of one month.

### Data analysis

IBM SPSS was used to clean the data and to obtain descriptive statistics. The data was analysed with Mplus version 8.3 [27]. We conducted a literature search for previous studies that examine the evidence for the factor validity of the selected measurement instruments and identified alternative models that were fitted for some of the measurement instruments. The theorized models, together with the alternative models, were tested for the respective measurement instruments. We applied CFA, bifactor CFA, exploratory structural equation modeling (ESEM), and bifactor ESEM to the AFM-2 (Study 1), ATQ-P (Study 2), and PHQ-9 (Study 4). Only CFA was applied to the GSEs (Study 3) and the SWLS (Study 5) since these measurement instruments consist of a single factor only and no cross-loadings and, or a general factor can be specified.

CFA is based on the independent clusters model, where it is assumed that each indicator only loads on a single latent (target) factor [28], where the cross-loadings on nontarget factors are also assumed to be zero. CFA is applied to evaluate how well the measured variables represent the number of constructs and to confirm or reject the measurement theory [28, 29]. CFA, however, fails to account for two important sources of construct-relevant psychometric multidimensionality, namely, the hierarchical nature of psychological constructs (that explains the associations of items to their specified target factors, as well as a higher-order factor, such as overall positive mental health), and the imprecise nature of items where items typically load on their target factors and have cross-loadings on nontarget but conceptually-related factors [29, 30, 31].

The first and second sources of construct-relevant multidimensionality are addressed by bifactor modeling [32] and ESEM [33], respectively. Bifactor models (e.g., the bifactor CFA model) permit the simultaneous specification of a general factor as well as separate orthogonal group factors which result from variance not explained by the general factor for different content domains [34]. A bifactor CFA model allows for the separation of the variance explained by the specific and general factors and tests if there is a general underlying factor that directly influences the items in addition to the influence of the specific factors [29, 35]. ESEM, on the other hand, integrates the characteristics of exploratory factor analysis (EFA) and CFA by allowing items to load on their intended factor(s), as well as on nonintended factors [28, 33]. ESEM models have been shown to produce better fit and more accurate factor correlations compared to CFA [36]. A bifactor ESEM model combines bifactor modeling and ESEM by concurrently allowing items to load on all factors (with nontarget loadings as close to zero as possible) and to load on both general and specific factors [29].

The robust maximum likelihood (MLR) estimator was applied to accommodate any deviations from normality for all five measurement instruments. The weighted least square mean and variance adjusted (WLSMV) estimator was additionally applied to the two measures (GSEs [Study 3] and PHQ-9 [Study 4]) that have only four response options [37]. We parameterized the CFA and bifactor CFA models by fixing the variances of the latent factors to one [38]. Oblique target rotation [33] and orthogonal bifactor target rotation [29] were applied for ESEM and bifactor ESEM, respectively. For the target rotation, all loadings on the nontarget factors were set to approximately, but not constrained to, zero [33]. There were no missing data.

We considered the guidelines postulated by Byrne [39] to determine how well each model fit the data. Comparative fit index (CFI) and Tucker-Lewis Index (TLI) values of .90 and larger indicate reasonable fit and values of .95 and larger indicate good fit. For both the standardized root mean square residual (SRMR, only applicable where the MLR estimator was applied) and the root mean square error of approximation (RMSEA), values below .08 suggest reasonable fit and values below .05 indicate good fit. A cut-off value of less than 1.0 suggest good fit for the weighted root mean square residual (WRMR), which is applicable only where the WLSMV estimator was applied [40]. Given that the chi-square test is highly sensitive to sample size, the CFI, TLI, RMSEA, and the SRMR or WRMR were considered in decision making. We examined the loadings of all items that made up a particular factor in determining if that factor is well-defined. Factor loadings higher than ±.40, preferably, and ±.30 at minimum were deemed indicative of a well-defined factor [41]. We inspected the size and significance of the loadings of the items on their respective factors and reported statistically significant cross-loadings on nontarget factors for measurement instruments where ESEM and bifactor ESEM analyses were conducted. We also interpreted small *R*^*2*^*-*values (percentage of the item variance explained by the model) and small factor loadings on their target factors as possible indications of local misfit.

We estimated the model-based omega coefficients of composite reliability [42] using the formula stipulated by Sánchez-Oliva et al. [43], where ω = (Σ|λ_i_|)^2^/ ([Σ|λ_i_|]^2^ + Σδ_ii_), with λ_i_ representing the factor loadings and δ_ii_ the error variances. Our interpretation of reliability values for first order (non-bifactor) models is based on the standard convention that satisfactory reliability estimates should be .70 and above [44]. Perreira et al. [45] suggest that omega reliability coefficients of .50 and above are satisfactory for bifactor models.

### Ethical considerations

The Health Research Ethics Committee (HREC) of the North-West University (NWU-00109-17-S1), South Africa, and the Noguchi Memorial Institute for Medical Research Institutional Review Board (NMIMR-IRB) of the University of Ghana (NMIMR.IRB CPN OO7/17-18), Ghana, approved this study. Permissions were also granted by the Regional and District Health Directorates of the SWD and the chiefs and elders of the communities involved. Participants were assured of the confidentiality of data and about their right to withdraw from the study up to the point of data analysis, without any consequences. Written informed consent was obtained from all participants.

## Results

### Demographic profile of participants

The demographic profile of the participants is shown in Table 1. The majority of participants were male, married, and had no formal education. All participants were fluent in Twi.

**Table 1:** Demographic profile of participants.

Variable	*n*	%
Gender		
*Male*	240	54.1
*Female*	204	45.9
Age	*Mean* = 37.9 (*SD* = 11.4; Range = 18–76)	
Marital Status		
*Single*	55	12.4
*Married*	326	73.4
*Cohabiting*	14	3.2
*Divorced/Separated*	25	5.6
*Widowed*	24	5.4
Education		
*None*	205	46.2
*Primary*	78	17.6
*Junior High*	85	19.1
*Senior High*	41	9.2
*Tertiary*	8	1.8
*Other*	2	6.1

### Superior model, factor loadings, and their reliabilities

The fit indices for all models are presented in Table 2. The standardized factor loadings for the superior model fit for all measurement instruments are displayed in Table 3.

**Table 2:** Fit statistics of models.

Model	χ^2^	*df*	*p*	CFI	TLI	RMSEA	90% CI	SRMR	WRMR
**AFM2**									
*1-factor CFA* (AFM-Model 1)	807.66	170	< .001	.714	.681	.094	.088, .101	.117	
*2-factor CFA* (AFM-Model 2)	*PSI not positively definite*								
*2-factor bifactor CFA* (AFM-Model 3)	*Theta not positively definite*								
*2-factor ESEM* (AFM-Model 4)	315.57	151	< .001	.926	.907	.051	.043, .059	.043	
*2-factor bifactor ESEM* (AFM-Model 5)	240.78	133	< .001	.952	.931	.044	.035, .053	.033	
**ATQ-P**									
*1-factor CFA; 30 items* (ATQ-P-Model 1)	1431.31	405	< .001	.858	.847	.077	.073, .082	.055	
*4-factor CFA; 30 items* (ATQ-P-Model 2)	*PSI not positively definite*								
*4-factor bifactor CFA; 30 items* (ATQ-P-Model 3)	*No convergence*								
*4-factor ESEM; 30 items* (ATQ-P-Model 4)	669.24	321	< .001	.952	.935	.051	.045, .056	.024	
*4-factor bifactor ESEM; 30 items* (ATQ-P-Model 5)	580.1	295	< .001	.961	.942	.048	.042, .054	.021	
*4-factor CFA; 22 items* (ATQ-Model 6)	665.90	203	< .001	.913	.901	.074	.067, .080	.049	
*4-factor bifactor CFA; 22items* (ATQ-P-Model 7)	*No convergence*								
*4-factor ESEM; 22 items* (ATQ-P-Model 8)	370.8	149	< .001	.958	.935	.059	.052, .067	.021	
*4-factor bifactor ESEM; 22 items* (ATQ-P-Model 9)	269.9	131	< .001	.974	.954	.050	.042, .059	.017	
*5-factor CFA; 22-items* (ATQ-Model 10)	590.59	199	< .001	.926	.914	.068	.062, .075	.047	
*5-factor bifactor CFA; 22 items* (ATQ-P-Model 11)	*No convergence*								
*5-factor ESEM; 22 items* (ATQ-P-Model 12)	269.9	131	< .001	.974	.954	.050	.042, .059	.017	
*5-factor bifactor ESEM; 22 items* (ATQ-P-Model 13)	237.1	114	< .001	.977	.953	.050	.041, .060	.016	
**GSEs**									
*1-factor CFA* (GSE-Model 1A)	161.58	35	< .001	.942	.925	.093	.078, .107	.028	
*1-factor CFA; WLSMV* (GSE-Model 1B)	312.77	35	< .001	.993	.991	.137	.123, .151		1.790
*1-factor CFA; item 4 w 5* (GSE-Model 2A)	199.90	34	< .001	.961	.948	.077	.063, .093	.025	
*1-factor CFA; item 4 w 5;* *WLSMV* (GSE-Model 2B)	220.94	34	< .001	.995	.994	.114	.100, .129		1.361
*1-factor CFA; item 4 w 5;* *2 w 3* (GSE-Model 3A)	100.91	33	< .001	.969	.957	.070	.055, .086	.024	
*1-factor CFA; item 4 w 5;* *2 w 3; WLSMV* (GSE-Model 3B)	205.50	33	< .001	.996	.994	.111	.097, .126		1.288
**PHQ-9**									
*1-factor CFA* (PHQ-Model 1A)	98.99	27	< .001	.909	.879	.079	.063, .097	.060	
*1-factor CFA; WLSMV* (PHQ-Model 1B)	344.84	27	< .001	.929	.905	.167	.152, .183		1.604
***Peterson et al*. *(2015)***									
*2-factor (CFA)* (PHQ-Model 2A)	94.97	26	< .001	.913	.879	.079	.063, .097	.060	
*2-factor CFA; WLSMV* (PHQ-Model 2B)	333.68	26	< .001	.931	.904	.167	.152, .184		1.576
*2-factor CFA; item 3 w 4* (PHQ-Model 3A)	57.94	25	< .001	.958	.940	.056	.037, .075	.051	
*2-factor CFA; item 3 w 4;* *WLSMV* (PHQ-Model 3B)	214.17	25	< .001	.958	.939	.134	.118, .151		1.249
*2-factor bifactor CFA* (PHQ-Model 4A)	*Data did not converge*								
*2-factor bifactor CFA;* *WLSMV* (PHQ-Model 4B)	*Data did not converge*								
*2-factor ESEM* (PHQ-Model 5A)	26.65	19	.113	.990	.982	.031	.000, .056	.022	
*2-factor ESEM; WLSMV* (PHQ-Model 5B)	58.28	19	< .001	.991	.983	.070	.050, .091		.568
*2-factor bifactor ESEM* (PHQ-Model 6A)	*Data did not converge*								
*2-factor bifactor ESEM;* *WLSMV* (PHQ-Model 6B)	*Theta not positively definite (item 2)*								
***Subotić et al*. *(2015);*** ***Krause et al*. *(2011)***									
*2-factor CFA* (PHQ-Model 7A)	89.40	26	< .001	.920	.889	.076	.059, .094	.056	
*2-factor CFA; WLSMV* (PHQ-Model 7B)	295.12	26	< .001	.940	.916	.157	.141, .173		1.475
*2-factor CFA; item 3 w 4* (PHQ-Model 8A)	58.81	25	< .001	.957	.938	.057	.038, .076	.050	
*2-factor CFA; item 3 w 4;* *WLSMV* (PHQ-Model 8B)	214.03	25	< .001	.958	.939	.134	.118, .151		1.244
*2-factor bifactor CFA* (PHQ-Model 9A)	*Data did not converge*								
*2-factor bifactor CFA;* *WLSMV* (PHQ-Model 9B)	*PSI not positively definite*								
*2-factor ESEM* (PHQ-Model 10A)	26.65	19	.113	.990	.982	.031	.000, .056	.022	
*2-factor ESEM; WLSMV* (PHQ-Model 10B)	58.28	19	< .001	.991	.983	.070	.050, .091		.568
*2-factor bifactor ESEM* (PHQ-Model 11A)	*Data did not converge*								
*2-factor bifactor ESEM;* *WLSMV* (PHQ-Model 11B)	*Theta not positively definite (item 2)*								
**SWLS**									
*1-factor CFA*	45.07	5	< .001	.941	.882	.138	.103, .176	.053	
*1-factor CFA; item 4 w 5*	12.02	4	< .001	.988	.970	.069	.026, .115	.014	

*Note*: χ^2^ = Chi-square test statistic; CFA = Confirmatory factory analysis; CFI = Comparative fit index; *df* = Degrees of freedom; ESEM = Exploratory structural equation modelling; *p* = Probability value; RMSEA = Root mean square error of approximation; TLI = Tucker-Lewis index; SRMR = Standardised root mean square residual; WLSMV = Weighted least square mean and variance adjusted; WRMR = Weighted root mean square residual; 90% CI = 90 percent confidence interval of the RMSEA

**Table 3:** Standardised factor loadings for models of best fit.

Item and Intended Subscale						*R*^*2*^
**AFM-2**	**AFM-2-T**	**PA**	**NA**			
1. Life on the right track, Co	.66**	**-.20**	.32			.48
2. Left alone, SS	-.25**	-.14	**-.01**			.17
3. Do whatever want, F	.63**	**-.04**	.50			.42
4. Thinking creatively, TC	.77**	-.11	**.30**			.590
5. Failure, SF	-.27**	**.03**	.60			.36
6. Nothing fun, Ch	-.29**	.07	**.39**			.34
7. Like yourself, SE	.72**	**.09**	.04			.54
8. Can’t be bothered, E	.23**	.49	.63*			.33
9. Close to people, SI	.75**	**-.08**	.61			.57
10. Best years are over, O	**-.09**	.01	**.59**			.36
11. Future looks good, O	.82**	**.10**	-.10			.67
12. Lost interest, SI	-.20**	-.05	.07**			.45
13. Energy to spare, E	.60**	**.03**	.54			.37
14. Change life, Co	.81**	-.42	-.09**			.70
15. Smiling a lot, Ch	.23**	-.04*	.07			.43
16. Thoughts useless, TC	**-.09**	-.20	**.03**			.42
17. Handle problems, SF	.67**	**.20**	.03			.43
18. Life stuck, F	-.14**	.13	.32**			.41
19. Loved and trusted, SS	.72**	**.12**	.01			.52
20. Something wrong, SE	**.04**	.28	.06**			.23
**ATQ-P**	**ATQ-P-T**	**DF**	**SE**	**OE**	**FE**	
1. Respected by my peers, OE	.77**	-.04	.07	.21*	.40	.64
2. Good sense of humour, OE	.81**	-.01	.04	**.16**	.46	.69
3. Future looks bright, FE	.80**	-.03	.39	.23	-.01**	.81
4. Successful, FE	.77**	.33	.41	.19	.01**	.81
5. Fun to be with, OE	.83**	-.01	.16	**.01**	.09	.76
6. Great mood, DF	.77**	-.15**	.24	.04	.01	.64
7. People care about me, DF	.74**	**-.02**	.06	.04	-.01	.55
10. Have good qualities, SE	.86**	-.07	**-.02**	.04	.06	.77
11. Comfortable with life, DF	.78**	-.02**	.06	-.11	0.03	.76
12. Good way with others, OE	.82**	.08	.07	**-.24**	-.10	.75
13. Lucky person, DF	.69**	-.08**	-.06	-.05	.06	.53
14. Friends support me, DF	.50**	.55**	-.06	.07	.01	.39
15. Life is exciting, DF	.80**	-.01*	-.01	-.18	-.06	.74
17. Social life is terrific, DF	.85**	**.66**	-.08	.20	-.11	.78
19. I’m so relaxed, DF	.68**	.25**	.15	-.11	-.11	.55
20. Life is running smoothly, DF	.62**	.01**	.13	.09	-.01	.83
21. Happy with my look, SE	.78**	.21	**-.11**	.01	-.05	.77
22. Take care of myself, SE	.77**	.35	-.13**	-.04	.08	.75
23. Deserve the best in life, SE	.76	.20	.02**	.06	.06	.71
25. Have useful qualities, SE	.87	.30	**-.09**	-.04	.01	.78
28.State opinions, SE	.72	.06	.01*	.01	.07	.58
29. My life getting better, DF	.62	**.20**	.05	.06	-.02	.72
**GSEs**	**GSEs-T**					
1. Solve difficult problems	.88**					.77
2. Get what I want	.80**					.63
3. Stick to aims	.84**					.71
4. Efficient	.86**					.75
5. Handle unforeseen situations	.84**					.71
6. Solve most problems	.92**					.68
7. Remain calm	.86**					.74
8. I can find solutions	.87**					.76
9. I can think of a solution	.83**					.85
10. Handle whatever comes	.92**					.85
**PHQ-9**	**NS**	**SM**				
1. Little interest/pleasure, CA	**-.07**	.74				.16
2. Feeling down/depressed, CA	.24*	.72				.47
3. Sleeping too much/little, SM	.81	.23**				.59
4. Tired/little energy, SM	.68	-.05**				.56
5. Poor appetite or overeating, SM	.70	.37*				.55
6. Feeling bad/failure, CA	.71**	.08				.74
7. Trouble concentrating, CA	.05**	.17				.63
8. Moving/speaking slowly, CA	.05**	-.01				.48
9. Suicidal thoughts, CA	.59**	.43				.29
**SWLS**	**SWLS-T**					
1. Life close to ideal	.87**					.75
2. Excellent life conditions	.91**					.83
3. Satisfied with life	.60**					.26
4. Gotten important things in life.	.51**					.36
5. Would change no part of my life	.87**					.76

*Note*: Target factor loadings are presented in bold. AFM-2 = Affectometer-2; AFM-T-2 = Affectometer-2 (Total scale); PA = Positive affect subscale; NA = Negative affect subscale; Co = Confluence; SI **=** Social interest; O = Optimism; F = Freedom; SE **=** Self-esteem; E **=** Energy; SF = Self-efficacy; Ch = Cheerfulness; SS **=** Social support; TC = Thought clarity; ATQ-P = Automatic thoughts questionnaire–positive; ATQ-P-T = Automatic thoughts questionnaire–positive (Total scale); DF = Daily functioning subscale; SE = Positive self-evaluations subscale; OE = Others’ evaluation of self subscale; FE = Positive future expectations subscale; GSEs = Generalized self-efficacy scale; GSEs-T = Generalized self-efficacy (Total scale); PHQ-9 = Patient health questionnaire-9; NS = Nonsomatic symptoms factor; SM = Somatic symptoms factor; SWLS = Satisfaction with life scale; SWLS-T = Satisfaction with life scale (Total scale); *R*^*2*^ = percentage variance explained by the factors;

*p < .05.

**p < .01.

### Study 1: Affectometer-2

#### Description of measure: Affectometer-2 (AFM-2)

The AFM-2 measures general happiness or a general sense of well-being by assessing the balance of positive and negative feelings in individuals’ recent experiences [46]. The short version, which is used in the present study, comprises two 10-item subscales: Positive Affect (AFM-PA) and Negative Affect (AFM-NA). The AFM-2 has five ordinal response levels (1 = *not at all;* 2 = *occasionally;* 3 = *some of the time*, 4 = *often;* 5 = *all of the time*). The total score, the positive-negative affect balance (PNB), is indicated by AFM-PA minus AFM-NA, where a higher score of AFM-PA over AFM-NA denotes positive mental health. The developers of the scale reported Cronbach’s alpha scores of .88 and .93 for the AFM-PA and AFM-NA, respectively [46]. Cronbach’s alpha scores of .64 and .79 for the AFM-PA and AFM-NA, respectively, were found in a Setswana student group in South Africa [47].

Similar to the original hypothesis [46], a one-factor solution was reported for a Scottish adult sample when principal component analysis (PCA) was conducted [48]. The authors, however, acknowledged the independence of positive and negative affect and cautioned that in some circumstances it might be useful to score the positive and negative subscales separately. On the contrary, a two-factor solution (AFM-PA and AFM-NA) was reported after CFA and EFA were applied to a dataset collated from a sample of Setswana-speaking South Africans [49].

#### Results and discussion

First, we tested the originally hypothesized [46] one-factor CFA model (AFM-Model 1). This model displayed a poor fit to the data (CFI = .714; RMSEA = 0.094). Next, we tested a two-factor CFA model (AFM-Model 2) with two correlated factors, namely, Positive Affect (AFM-PA; items 1, 3, 4, 7, 9, 11, 13, 14, 17, and 19) and Negative Affect (AFM-NA; items 2, 5, 6, 8, 10, 12, 15, 16, 18, and 20) as suggested by Wissing et al. [49]. The psi matrix was not positively definite for this model. We proceeded to test a two-factor bifactor CFA model (AFM-Model 3) with the two specified factors (AFM-PA and AFM-NA) and a general factor onto which all items loaded, with the specific factors being orthogonal. The theta matrix was not positively definite for this model. Consequently, we tested a two-factor ESEM model (AFM-Model 4). This model demonstrated reasonable fit (CFI = .926; RMSEA = 0.051). Subsequently, a two-factor bifactor ESEM model (AFM-Model 5) was tested. This model displayed superior fit (CFI = .952; RMSEA = 0.044) compared to the previous models.

For AFM-Model 5, ten of 20 items presented with strong and statistically significant loadings on the general factor. However, only one item (item 15: *“I wish I could change some part of my life”*) intended for the AFM-NA specific factor displayed strong and statistically significant loading. Items 8, 12, 18, and 20 presented weak but statistically significantly cross-loadings on the nontarget AFM-PA factor. Item 15 exhibited strong and statistically significant cross-loading on the nontarget AFM-PA factor. The majority of items intended for the AFM-PA specific factor (except items 4 and 14) displayed strong and statistically significant factor loadings. The omega coefficient for AFM-Model 5 was .88, .43, and .72 for the total scale, AFM-PA, and AFM-NA, respectively. Item 2 had an exceptionally small *R*^*2*^-value (.17), which means that the model explains only 17% of the variance contained within the item. All other items presented with *R*^*2*^-values ranging from .23 to .67.

As far as could be established, this is the first study to apply the bifactor ESEM model to the AFM-2. The application of bifactor ESEM helps to clarify the factor structure of the AFM-2 further and provides a deeper understanding of the nature of the complex interaction and role of negative and positive human experiences in mental health functioning. Previous studies [30, 35] report that bifactor models outperform and produce better fits when compared to traditional CFA and PCA analysis.

The findings of the present study are consistent with previous report by Wissing et al. [49], who applied CFA, PCA, and EFA to data from a sample of Setswana-speaking adults. The results are however inconsistent with previous research [46, 48] that reported a one-factor solution for the AFM-2 when CFA was conducted. The findings, generally, suggest that the total score of the Twi version and the AFM-NA subscale can be interpreted and used in subsequent research in this population. However, caution should be applied when interpreting the AFM-PA subscale scores for this group, given the low reliability value it presented. Given the new inclusive approach of mental health (i.e., the dual-systems model) that takes into account both the negative and positive attributes of individuals, valid, reliable, and culturally-adapted mental health measures, such as the AFM-2, that evaluate both positive and negative dimensions of the human experience are needed. Although the AFM-2 has been used for research across various contexts, there is no evidence supporting its factor validity, including the original English version, or in any native language in the Ghanaian setting. Considering its potential usefulness for holistic mental health assessment, the validation of the Twi version of the AFM-2 could facilitate the conduct of empirical research and the evaluation of mental health interventions in the current group. The Twi version demonstrated satisfactory reliability for the total scale and the AFM-NA subscale, with adequate model fit and can therefore be for used in this group.

### Study 2: Automatic Thoughts Questionnaire–Positive

#### Description of measure. Automatic thoughts questionnaire–positive (ATQ-P)

The ATQ-P is a 30-item self-report scale that assesses the frequency of positive thoughts [50]. Respondents rate each item on a 5-point Likert scale according to how frequently each thought or a similar thought has occurred to them during the past week (1 *= never*, 3 *= sometimes*, 5 *= all the time*). There are four subscales (i) Daily functioning (ATQ-P-D), (ii) Self-evaluation (ATQ-P-S), (iii) Others Evaluation of Self (ATQ-P-O), and (iv) Future Expectations (ATQ-P-F). A total positive automatic thoughts score (ATQ-P-T) is calculated by adding the scores of all the items together. The ATQ-P-T demonstrated a high level of internal consistency with a Cronbach’s alpha value of .96 among a sample of American adolescents [51]. A Cronbach’s alpha value of .94 was also reported for the Dutch version in an adult Dutch population [52].

Previous studies provided support for three different models of the ATQ-P. Jolly et al. [51] reported a four orthogonal factor solution similar to the original factor structure hypothesized by Ingram et al. [50]. Bryant and Baxter [53] also reported a four-factor solution with factors comparable to the original hypothesis. However, contrary to the developers’ interpretation, Bryant and Baxter postulated that the four factors are negatively correlated, rather than orthogonal. A decade later, Boelen [52] reported a five oblique factor model among a bereaved American adult sample. Boelen’s model was a slight modification of the original four orthogonal factor model, but with an added factor, the *Positive Social Functioning* (ATQ-P-SF), drawn from items of the ATQ-P-D factor.

#### Results and discussion

We started by fitting a one-factor 30-item CFA model (ATQ-P-Model 1). The results indicated a poor fit (CFI = .858; RMSEA = 0.077). We proceeded to test the four-factor 30-item CFA model (ATQ-P-Model 2) suggested by Jolly et al. [51], with the factors specified as ATQ-P-D (items 3, 6, 7, 8, 11, 13, 14, 15, 17, 19, 20, 29); ATQ-P-S (items 16, 18, 21, 22, 28); ATQ-P-O (items 1, 2, 5, 10, 12) and ATQ-P-F (items 4, 9, 23, 24, 25, 26, 27, 30). The psi matrix was not positively definite for this model. Consequently, we tested a four-factor 30-item bifactor CFA model (ATQ-P-Model 3) based on the structure proposed by Jolly et al. [51]. This model failed to converge. Next, we tested a four-factor 30-item ESEM model (ATQ-P-Model 4) based on the structure suggested by Jolly and colleagues. The results displayed reasonable fit indices (CFI = 0.952; RMSEA = 0.051), but with lower fit indices when compared to the four-factor 30-item bifactor ESEM model (CFI = 0.961; RMSEA = 0.048; ATQ-P-Model 5) that was subsequently tested.

Next, a four-factor 22-item CFA model (ATQ-Model 6) suggested by Bryant and Baxter [53], with factors comparable to the four-factor 30-item CFA model originally hypothesized by Ingram and Wisnicki [50] was investigated. This model displayed acceptable fit (CFI = .913; RMSEA = 0.074), but with lower fit indices when compared to ATQ-P-Model 5. The four-factor 22-item bifactor CFA model which was subsequently tested (ATQ-P-Model 7) failed to converge. We proceeded to test a four-factor 22-item ESEM model (ATQ-P-Model 8, CFI = 0.958; RMSEA = 0.059). This model showed improved fit when compared to ATQ-P Model 6, but was outperformed by the four-factor 22-item bifactor ESEM model (ATQ-P-Model 9, CFI = 0.974; RMSEA = 0.050) that was subsequently tested.

We proceeded to test Boelen’s [52] five-factor 22-item CFA (ATQ-P-Model 10), with the factors specified as: ATQ-P-D (items 6, 11, 13, 15, 19, 20, 29); ATQ-P-S (items 10, 21, 22, 23, 25, 28); ATQ-P-O (items 1, 2, 5, 12); ATQ-P-F (3, 4); and Positive Social Functioning (ATQ-P-SF; items 7, 14, 17). This model displayed a reasonable fit to the data (CFI = .926; RMSEA = 0.068). The five-factor 22-item bifactor CFA model (ATQ-P-Model 11) that was consequently evaluated failed to converge. Next, we tested a five-factor 22-item ESEM model (ATQ-P-Model 12, CFI = 0.974; RMSEA = 0.050). This model outperformed ATQ-P Model 10. Lastly, we tested a five-factor 22-item bifactor ESEM model (ATQ-P-Model 13). This model displayed good fit to the data (CFI = 0.977; RMSEA = 0.051), but with a larger RMSEA index compared to ATQ-P-Models 9 and 12.

Overall, the four-factor 22-item bifactor ESEM (ATQ-P-Model 9) and the five-factor 22-item ESEM (ATQ-P-Model 12) models displayed superior model fit, with similar fit indices. Almost all items presented with strong and statistically significant loadings on their general target factors for both models. The ATQ-P-Model 9 presented with a very high reliability value for the total scale (.98) and satisfactory reliability values for the ATQ-P-D (.67), ATQ-P-S (.54), and the ATQ-P-O (.66) subscales, but not for the ATQ-P-F (.35) subscale. Similarly, the ATQ-P-Model 12 presented satisfactory reliability values for four subscales: .88 (ATQ-P-D), .87 (ATQ-P-S), .80 (ATQ-P-O), .86 (ATQ-P-F), but a very low value of .05 for the ATQ-P-SF. Since the four-factor 22-item bifactor ESEM model displayed a low reliability value for the ATQ-P-F subscale (.35 out of .50 for bifactor models) compared with the very low value (.05 out of .70 for non-bifactor models) for the ATQ-P-SF subscale of the five-factor 22-item ESEM model, the factor loading and *R*^*2*^ statistics are presented for the four-factor 22-item bifactor ESEM model. For the four-factor 22-item bifactor ESEM model, four (items 11, 14, 20, and 29) of ten items of the ATQ-P-D factor, three (items 22, 23, and 28) of six items of the ATQ-P-S factor, and both items (items 3 and 4) of the ATQ-P-F factor displayed strong and statistically significant factor loadings on their target factors. None of the items intended to assess ATQ-P-O loaded significantly on their hypothesized target factor or demonstrated any significant cross-loadings on another specific factor. Only item 21 (“*I’m happy with the way I look*”) displayed strong and statistically significant cross-loading on the nontarget ATQ-P-D factor. Notably, all items displayed high *R*^*2*^-values ranging from .39 to .83.

The results from the current study suggest that the items on the ATQ-P cluster into either four dimensions of positive thinking (*individual’s daily functioning*, *self-evaluation*, *others’ evaluation of self*, *future expectations*, *and social relationships*), or into five dimensions, with an additional dimension (*positive social functioning*) in the present sample. The reliability values in the current sample (e.g., omega coefficients between .80 and .88 for four of the five subscales of the ATQ-P-Model 12) are comparable to Boelen’s [52] reported reliability values (.78 to .88), although the ATQ-P-PF subscale for ATQ-P-Model 12 presented with a very low reliability value (.05) for which it should not be interpreted or used to draw conclusions in the current sample.

With an increasing recognition that the human experience comprises negative and positive dimensions, the availability of a locally validated version of the ATQ-P scale could facilitate the study of the roles that positive thoughts play in people’s psychological functioning [51–53]. For instance, it is well established that languishing (i.e., low or absence of positive mental health) is a predisposition to psychopathology [9, 10]. A culturally-adapted version of the ATQ-P may also be useful in epidemiological surveillance to assess positive human experiences and to evaluate positive thinking patterns of patients under treatment. We found evidence for the factor validity for the Twi version of the ATQ-P in the current Ghanaian sample. However, on the basis of the low reliability scores of the ATQ-P-F and the ATQ-P-PF subscales, it is recommended that scores of these subscales should not be interpreted for the current group.

### Study 3: Generalized Self-Efficacy Scale

#### Description of measure. Generalized self-efficacy scale (GSEs)

The GSEs is a 10-item self-report measure that assesses optimistic self-beliefs to cope with a variety of difficult demands in life [54]. Responses to items are measured on a 4-point scale ranging from 1 (*not true at all*) to 4 (*exactly true*). The total score is calculated as the sum of all items and ranges between 10 and 40, with higher scores indicating more self-efficacy. Urban [55] reported a high reliability coefficient (α = .90) among a sample of multicultural university students comprising black Africans, Indians, and white South Africans. Löve et al. [56] also established evidence for the validity of GSEs, with a high Cronbach’s alpha value of .90 in an adult Swedish sample.

Similar to the original hypothesis [54], a large body of research provides strong evidence that the GSEs represents a unidimensional construct [55–57], including a study that involved a large adult sample from 25 countries [58]. On the contrary, Zhou [59] reported a model with two distinct factors distinguished as c*oping self-efficacy* and *action self-efficacy* among a sample of Chinese university students when EFA was applied.

#### Results and discussion

We first tested a one-factor CFA model (GSEs-Model 1A) as originally hypothesized [54], using the MLR estimator. The result displayed poor fit (CFI = .942; RMSEA = 0.093). Given that the scale uses four response options, we also applied the WLSMV estimator (GSEs-Model 1B). Results showed improved fit when the CFI and TLI indices were considered (CFI = .993; TLI = .991). However, the RMSEA displayed a large value of 0.137, indicating poor fit. The analysis revealed a high residual correlation (modification index [MI] = 41.8) between item 4 (*“I am confident that I could deal efficiently with unexpected events”*) and 5 *(“Thanks to my resourcefulness*, *I know how to handle unforeseen situations”*) for GSEs Model 1. Consequently, we tested a one-factor CFA model with the residuals of items 4 and 5 correlated using the MLR (GSEs-Model 2A) and the WLSMV (GSEs-Model 2B) estimators. GSEs-Model 2A (CFI = .961; RMSEA = 0.077) and GSEs-Model 2B (CFI = .995; RMSEA = 0.114) presented improved fit indices compared to GSEs-Models 1A and 1B. Items in GSEs-Models 2A and 2B also demonstrated high and significant factor loadings (.796 –.924) on the latent factor.

A high residual correlation between items 2 and 3 was suggested (MI = 21.0) for GSEs-Model 2A. Consequently, a one-factor CFA model using the MLR (GSEs-Model 3A) and the WLSMV (GSEs-Model 3B) estimators with the residuals of items 4 and 5, as well as items 2 and 3 correlated was investigated. The results displayed good fit indices for GSEs-Model 3A (CFI = .969; RMSEA = 0.070) but not for the GSEs-Model 3B (CFI = .996; RMSEA = 0.111), with a large RMSEA index. Overall, the GSEs-Model 2A displayed superior model fit, with a high reliability value of .97. We found very strong and statistically significant loadings on the total generalized self-efficacy factor, with high *R*^*2*^-values (.63 –.85).

A possible explanation for the high correlation between items 4 and 5 is that, theoretically, both items appear to refer to an individual’s appraisal of his or her ability to handle future, unforeseen circumstances and events. Our findings are consistent with previous research [55–57] that provided strong evidence that the GSEs is underpinned by a unidimensional measurement model. Findings from a multinational study involving 25 countries also demonstrated that the one-factor structure is adequate at both the individual- and national-level [58]. On the contrary, Zhou [59] reported a two-factor structure model (distinguished as *coping self-efficacy* and *action self-efficacy*) for a sample of Chinese university students.

The present study is one of the few to provide evidence for the factor validity of the Twi-translated version of the GSEs among a nonclinical sample, and specifically, in an adult sample from a rural and resource-limited collectivistic community setting. Findings of the current study show that the factor structure and reliability value of the Twi version of the GSEs scale are generally consistent with previous studies, including those conducted among clinical population [56, 57]. Given its favourable reliability and acceptable model fit, the Twi version of the GSEs is recommended for use in the current sample to evaluate individuals’ optimistic self-beliefs.

### Study 4: Patient Health Questionnaire-9

#### Description of measure. Patient health questionnaire-9 (PHQ-9)

The PHQ-9 consists of nine items designed to assess the nine DSM-IV diagnostic criteria for depression [60]. Items are rated on a 4-point Likert scale specified as 0 (*never*), 1 (*several days*), 2 (*more than half of the days*), and 3 (*most days*). Total scores of 5, 10, 15, and 20 respectively represent mild, moderate, moderately severe, and severe depression [60]. The PHQ-9 was found to be valid with a Cronbach’s alpha value of .89 in a group of primary care patients previously diagnosed with depression [60]. Adewuya et al. [61] also reported a Cronbach’s alpha value of .85 in a Nigerian university student population.

Consistent with the original hypothesis [60], a number of studies [67, 68] reported a one-factor structure for the PHQ-9 across varied contexts and population. Nonetheless, a significant number of studies also reported a two-factor structure when CFA, EFA, and multilevel CFA (MCFA) were applied [62–66]. The two factors were either designated as *somatic* and *affective* [62–65] or *somatic* and *cognitive/affective* [66].

#### Results and discussion

We started by fitting a one-factor CFA model (PHQ-Model 1A) to the data, as was originally proposed [60], using the MLR estimator. As shown in Table 2, the PHQ-9 Model 1 displayed a reasonable fit (CFI = .909; RMSEA = 0.079). Since the PHQ-9 has four response options, we also applied the WLSMV estimator (PHQ-Model 1B). The results indicated an improved CFI (CFI = .929), but poor RMSEA (RMSEA = 0.167) index.

We proceeded to evaluate the two-factor CFA model suggested by Petersen et al. [65] with two correlated factors (*somatic symptoms* [PHQ-9-S; items 3, 4, 5, 7, and 8]; *nonsomatic/affective symptoms* [PHQ-9-N; items 1, 2, 6, and 9]) using the MLR (PHQ-Model 2A) and the WLSMV (PHQ-Model 2B) estimators. PHQ-Model 2A (CFI = .913; RMSEA = 0.079) displayed reasonable fit, but not PHQ-Model 2B (CFI = .931; RMSEA = 0.167), considering its RMSEA index. A high residual correlation (MI = 37.1) between item 3 (*“Trouble falling or staying asleep/ OR sleeping too much”*) and 4 (*“Feeling tired/ having little energy”*) was suggested for PHQ-Model 2A. Consequently, we specified residual correlations between items 3 and 4 using the MLR (PHQ-Model 3A) and the WLSMV (PHQ-Model 3B) estimators. The fit improved for PHQ-Model 3A (CFI = .958; RMSEA = 0.056) as well as for the PHQ-Model 3B (CFI = .958; RMSEA = 0.134).

We further applied bifactor CFA, ESEM, and bifactor ESEM to the two-factor model proposed by Petersen et al. [65] using the MLR and WLSMV estimators (see Table 2: PHQ-Model 4A –PHQ-Model 6B). Whereas the two-factor bifactor CFA models (PHQ-Model 4A and 4B) and two-factor bifactor ESEM models (PHQ-Model 6A and 6B) failed to converge for both estimators, the two-factor ESEM model displayed good fit for the MLR estimator (CFI = .990; RMSEA = 0.031; PHQ-Model 5A) and an improved fit (CFI = .991; RMSEA = 0.070; PHQ-Model 5B) for the WLSMV estimator. Overall, the PHQ-Model 5A displayed superior fit for the model suggested by Peterson et al. [65], with a satisfactory reliability value of .78 for the PHQ-9-S subscale and a low value of .48 for the PHQ-9-N subscale. All five items of the PHQ-9-S subscale and two of four items of the PHQ-9-N subscale displayed strong and statistically significant factor loading to their intended factors.

Furthermore, we tested the two-factor CFA model with two correlated factors (*somatic symptoms* [items 3, 4, and 5] and *nonsomatic/affective symptoms* [items 1, 2, 6, 7, 8, and 9]) suggested by Krause et al. [64] and Subotić et al. [66]. The results indicated reasonable fit when the MLR estimator was applied (CFI = .920; RMSEA = 0.076; PHQ-Model 7A) but poor fit with the WLSMV estimator (CFI = .949; RMSEA = 0.157; PHQ-Model 7B), considering the RMSEA index. Following a high residual correlation (MI = 31.7) between items 3 and 4 from the preceding analysis, we specified residual correlations between items 3 and 4 using the MLR (PHQ-Model 8A) and the WLSMV (PHQ-Model 8B) estimators. The fit indices improved considerably for PHQ-Model 8 (CFI = .957 and RMSEA = 0.057) and for PHQ-Model 8B (CFI = .958; RMSEA = 0.134). We continued to apply the MLR and WLSMV estimators in turns to test the fit of two-factor bifactor CFA, ESEM, and bifactor ESEM models (see Table 2: PHQ-Model 9A –PHQ-Model 11B). The two-factor bifactor CFA and the two-factor bifactor ESEM models did not converge. The two-factor ESEM model displayed good fit when the MLR estimator was applied (CFI = .990; RMSEA = 0.031; PHQ-Model 10A) and an improved fit for the WLSMV estimator (CFI = .991; RMSEA = 0.070; PHQ-Model 10B). For the two-factor structure proposed by Krause et al. [64] and Subotić et al. [66], the two-factor ESEM model (PHQ-Model 10A) demonstrated superior fit, with satisfactory omega coefficients of .76 for the PHQ-9-S subscale, and a modest reliability value of .69 for the and PHQ-9-N subscale. It is noted that both models (PHQ-Model 5A and PHQ-Model 10A) presented similar fit indices.

Given that the two-factor ESEM model (PHQ-Model 10A) had larger reliability values for the subscales compared to PHQ-9 Model 5A (.76 and .69 vs .78 and .48), its factor loadings and related statistics are presented. Four of six items (items 6, 7, 8, and 9) and two of three items (items 3 and 4) displayed strong and statistically significant loadings on their intended *cognitive-affective* and *somatic* factors, respectively. Notably, item 5 (“*Poor appetite or overeating*”) presented a strong and statistically significant cross-loading on the nontarget *cognitive-affective* factor. Similarly, item 1 (*“Little interest/pleasure in doing things”*) and 2 (*“Feeling down/depressed/hopeless”*) displayed a strong and statistically significant cross-loading on the nontarget *somatic* factor. Items 1 and 2 presented exceptionally small *R*^*2*^-values of .16 and .29 respectively, suggesting that the model explained only 16% and 29% of the variance contained within the item 1 and 2, respectively. All other items presented with adequate *R*^*2*^-values (.47–.74).

The findings of the present study are consistent with previous research [63, 65, 66], although some other studies [67, 68] also reported a unidimensional structure. For the current sample, the PHQ-9 can be subdivided into *somatic* (PHQ-9-S) and *nonsomatic/affective* (PHQ-9-N) factors, which is comparable to previous reports in clinical [63–65] and nonclinical [62, 66] samples. We noted that items 7 and 8 loaded on the *somatic* factor in the model suggested by Peterson et al. [65], and instead, on the *nonsomatic/affective* factor in the model recommended by Krause et al. [64] and Subotić et al. [66]. A possible explanation for the behavior of these items could be that although both items (Item 7: *“Trouble concentrating”*; and Item 8: *“Being so fidgety or restless”*) are behavioristic in nature, they could be driven and reinforced by some emotions [69]. The reliability values found in the current sample (.76 and .69) are comparable to previous report among a sample of university students in Nigeria [70] and in the United States [71].

Although the PHQ-9 is a widely validated tool, the current study is among the first to apply a bifactor analysis to test its structure in the African context. In spite of the recent increase in the use of the PHQ-9 in epidemiological surveillance in Ghana, only one study [72] has examined the psychometric properties of the original English version of the PHQ-9 among a sample of Senior High School students in Ghana. The recent advocacy for the integration of mental health into primary health care to allow for comprehensive assessment and treatment of mental disorders, particularly in LAMIC [73], necessitates valid and reliable measures that are also culturally sensitive and has the ability to detect the presence of mental illness in nonclinical populations. Overall, our findings suggest that depression should be understood, for the current sample, as characterized by somatic and nonsomatic or affective structure, rather than as an expression of unidimensional structure. In the present study, the Twi version of the PHQ-9 showed adequate reliability and model fit and can be administered to evaluate self-reported levels of depression in the current sample. Caution should however be applied when interpreting the *nonsomatic* factor scores for this population, considering the low reliability score it presented.

### Study 5: Satisfaction with Life Scale

#### Description of measure. Satisfaction with life scale (SWLS)

Designed to assess a person’s global judgment of life satisfaction, the 5-item SWLS theoretically evaluates an individual’s life circumstances in comparison to his or her standards [74]. Responses are on a 7-point Likert scale, where 1 = *strongly disagree* and 7 = *strongly agree*. Total scores range from 5 to 35, where higher scores (31–35) indicate greater life satisfaction. Wissing and van Eeden [75] provided evidence for the validity of the SWLS with modest reliability (α = .67) among a multicultural sample of South African adults. Diener et al. [74] reported a one-factor solution for the SWLS in the original validation study. This has since been replicated across several populations and contexts [49, 75] when CFA, EFA, and PCA were applied.

#### Results and discussion

First, we evaluated the one-factor CFA model (SWLS-Model 1) as originally hypothesized [74]. This model resulted in a poor fit (CFI = .941; RMSEA = 0.138). An MI value of 33.9 suggested a high residual correlation between item 4 *(“So far I have gotten the important things I want in life”*) and 5 (*“If I could live my life over*, *I would change almost nothing”*). Consequently, a one-factor CFA model with the residual correlations between items 4 and 5 specified (SWLS-Model 2) was examined. The SWLS-Model 2 (CFI = .988; RMSEA = 0.069) presented superior fit and outperformed SWLS-Model 1. An omega coefficient of .87 was found for SWLS-Model 2. All five items presented strong and statistically significant factor loadings. Of note, item 5 presented with a comparatively low, but statistically significant factor loading (.51). In this model, item 5 displayed a low *R*^*2*^-value (.26), indicating that only 26% of the variance contained within the item is explained by the model.

In line with previous studies, a one-factor CFA model (residuals of items 4 and 5 correlated) demonstrated superior model fit. This finding is consistent with previous validation studies that also reported a one-factor CFA model for adult [49, 75] and adolescent [76] samples. We found a high modification index between items 4 and 5, similar to previous reports [77]. A possible explanation for this correlation is that both items refer to an individual’s present state of satisfaction with life with respect to the past. To the contrary, items 1–3 pertain to satisfaction with present life. The analysis revealed, similar to findings from previous studies [49, 76], that item 5 presented a relatively low, but statistically significant factor loading. In the present study, the reliability value for the SWLS was .87.

The findings from the current study provide additional evidence in support of previous studies confirming the single-factor structure of the SWLS [49, 74–76]. For the current sample, the findings suggest that the scale has satisfactory reliability and sufficient model fit similar to the results from the original scale development study [74] and can be administered to evaluate the level of satisfaction with life in this group.

## General discussions. strengths, limitations, and future directions

This paper set out to explore the evidence for the factorial validity of the Twi-translated versions of five mental health and well-being measurement instruments, namely the AFM-2, ATQ-P, GSEs, PHQ-9, and the SWLS, with data from a sample of adult residents of four rural poor communities in Ghana. The findings in this study provide evidence that the Twi versions of all five mental health and well-being scales, particularly the total scales, are valid and reliable with sufficient model fits and can be used in basic research and interventional studies in the current sample. The major strengths of the present study include the application of both traditional and recently developed models and techniques in the analyses of the data. An additional strength of this study is the target population. Unlike previous studies, the sample in the current study is composed of rural adults who were also non-English speakers. Whereas this study was a first attempt to establish the evidence for the validity of the Twi versions of these five measures of mental health and well-being in the Ghanaian context, more of such validation studies are needed to provide researchers and practitioners with valid and reliable tools for research and to evaluate the effectiveness of mental health interventions in the target population. Such efforts would also broaden our understanding of how these constructs manifest in the current, understudied context.

Although this study contributes to the literature, as the first, to the best of our knowledge, to provide information on the reliability and the factor structure of the Twi versions of these selected mental health and well-being measures in the Ghanaian setting, we acknowledge the following limitations. Firstly, our participants, on average, were middle-aged, had little or no formal education, and were economically disadvantaged. These unique characteristics of our sample may limit the generalisability of the findings. Secondly, the current sample was mostly non-English speakers and comprises community members who were largely of the Akan heritage–who can also be described as a more collectivistic group. Future studies should include English-speaking samples from urban settings for a comparative analysis of the results. Furthermore, given the cultural diversity in Ghana, it would be important for future studies to include samples from different regions with different cultural heritages and orientations for a better representation. Lastly, while our sample size was adequate to perform the intended analyses, it was too small to perform measurement invariance analyses. We recommend that future studies should include larger sample sizes to permit measurement invariance analysis and thereby provide additional psychometric information of the measurement instruments involved. We wish to emphasize, however, that, the main purpose of this study was to provide evidence for the factorial validity of the selected measurement instruments. We recommend that future studies should investigate other forms of validity, such as criterion-related validity and apply other analyses such as test-retest reliability to further examine the psychometric properties of the measurement instruments, perform in-depth item-analysis for the individual scales.

## Conclusions

The availability of reliable and valid measures, such as the present Twi-translated versions of the AFM-2, ATQ-P, GSEs, PHQ-9, and the SWLS, can be valuable resource for mental health researchers and practitioners working to promote well-being and positive mental health in one of the most spoken Ghanaian languages in the context of the current sample. The results in this study showed that the total scales of all five scales and some subscales attained satisfactory reliability values with acceptable model fit, but that further research is necessary to explore some problematic items, especially in the case of the AFM-PA and ATQ-P-F subscales. It is our hope that the availability of these Twi translated versions of these five mental health and well-being measures will afford researchers the opportunity to the conceptualize and evaluate diverse array of mental health, that is, positive mental health and mental ill(health) in this Ghanaian context. Additionally, these translated measuring instruments could serve as a valuable resource for researchers to facilitate the evaluation of the effects of mental health intervention programs in the current sample.

## Supporting information

S1 File (DOC)

## Abbreviations

AFM-2: Affectometer-2
AFM-NA: AFM (Negative Affect subscale)
AFM-PA: AFM (Positive Affect subscale)
ATQ-P: Automatic Thoughts Questionnaire–Positive
ATQ-P-D: ATQ-P (Daily functioning subscale)
ATQ-P-F: ATQ-P (Future expectations)
ATQ-P-O: ATQ-P (Others’ evaluation of self subscale)
ATQ-P-S: ATQ-P (Self-evaluation subscale
CFA: Confirmatory factor analysis
CFI: Comparative fit index
ESEM: Exploratory structural equation modeling
GSEs: General self-efficacy scale
HREC: Health Research Ethics Committee
MLR: Robust maximum likelihood
NMIMR-IRB: Noguchi Memorial Institute for Medical Research Institutional Review Board
NWU: North-West University
PHQ-9: Patient Health Questionnaire–9
PNB: Positive-negative affect balance
RMSEA: Root mean square error of approximation
SRMR: Standardized root mean square residual
SWD: Sunyani West District
SWLS: Satisfaction with Life Scale
TLI: Tucker–Lewis index
WLSMV: Weighted least square mean and variance adjusted
WRMR: Weighted root mean square residual

## References

[1] Bolier L, Haverman M, Kramer J, Westerhof GJ, Riper H, Walbur BE. Positive psychology interventions: a meta-analysis of randomized controlled studies. BMC Public Health. 2013;13(119). 10.1186/1471-2458-13-119 23390882 PMC3599475

[2] Weiss LA, Westerhof GJ, Bohlmeijer ET. Can we increase psychological well-being? The effects of interventions on psychological well-being: A meta-analysis of randomised controlled trials. PLoS ONE. 2016;11:1–16. 10.1371/journal.pone.0158092 PMC4915721 27328124

[3] Delle Fave A, Brdar I, Freire T, Vella-Brodrick D, Wissing MP. The eudaimonic and hedonic components of happiness: Qualitative and quantitative findings. Social Indicators Research. 2011;100:185–207.

[4] Mellins CA, Kauchali S, Nestadt DF, Bai D, Aidala A, Myeza N, et al Validation of the Client Diagnostic Questionnaire to Assess Mental Health in South African Caregivers of Children. Clinical Psychology and Psychotherapy. 2017;24:245–254. 10.1002/cpp.2008 26923182 PMC5002382

[5] Diener E, Oishi S, Lucas RE. Personality, culture, subjective well-being: Emotional and cognitive evaluations of life. Annual review of psychology. 2003;54:403–425. 10.1146/annurev.psych.54.101601.145056 12172000

[6] Ryan RM, Deci EL. On happiness and human potentials: A review of research on hedonic and eudemonic well-being. Annual review of psychology. 2001;52:141–166. 10.1146/annurev.psych.52.1.141 11148302

[7] Ryff CD, Keyes CLM. The structure of eudaimonic well-being revisited. Journal of Personality and Social Psychology. 1995;69:719–727. 10.1037//0022-3514.69.4.719 7473027

[8] Lomas T, Ivtzan I. Second wave positive psychology: Exploring the positive-negative dialectics of wellbeing. Journal of Happiness Studies. 2016;17:1753–1768.

[9] Wong PTP. Positive psychology 2.0: Towards a balanced interactive model of the good life. Canadian Psychology/Psychologie Canadienne. 2011;52:69–81. 10.1037/a0022511

[10] Schutte L, Wissing MP, Ellis SM. Problematic factorial validity of three language versions of the Basic Psychological Needs Scale (BPNS): Why and what are the implications? Journal of Happiness Studies. 2017;19:1175–1194. 10.1007/s10902-017-9861-2

[11] De Kock F, Kanjee A, Roodt G. Cross cultural test adaptation and translation In Foxcroft C., & Roodt G. (Eds). Introduction to psychological assessment in the South African context. 2013 South Africa: Oxford University Press.

[12] Idang GE. African culture and values. UNISA Phronimon. 2015;16:97–111.

[13] Oishi S. The psychology of residential mobility: Implications for the self, social relationships and well-being. Perspectives on Psychological Science. 2010;5:5–21. 10.1177/1745691609356781 26162059

[14] Glozah FN, Pevalin DJ. Factor structure and psychometric properties of the General Health Questionnaire (GHQ-12) among Ghanaian adolescents. Journal of Child and Adolescent Mental Health 2015;27:53–57. 10.2989/17280583.2015.1007867 25958797

[15] Glozah FN, Pevalin DJ. Psychometric Properties of the Perceived Social Support from Family and Friends Scale: Data from an Adolescent Sample in Ghana. Journal of Child and Family Studies.2017;26:88–100.

[16] Wilson A, Yendork JS, Somhlaba NZ. Psychometric Properties of Multidimensional Scale of Perceived Social Support among Ghanaian Adolescents. Child Indicators Research. 2016;3:1–15.

[17] Ghana Statistical Service. 2010 Population and Housing Census: National Analytical Report. Accra: Ghana Statistical Service. 2013. Retrieved from http://www.statsghana.gov.gh/docfiles/2010phc/National_Analytical_Report.pdf

[18] Ghana’s District League Table (2018). 2018/19 District League Table II with new perspectives and modified methodology. Retrieved from https://www.unicef.org/ghana/media/2131/file/2018-2019-The-District-League-Table-II.pdf

[19] Ghana Statistical Service (GSS), Ghana Health Service (GHS), and ICF International. Ghana Demographic and Health Survey 2014. Rockville, Maryland, USA: GSS, GHS, and ICF International. 2015.

[20] World Bank. Ghana Country Overview. 2017b. http://www.worldbank.org/en/country/ghana/overview

[21] Bonthuys A, Botha KFH, Nienaber AW, Fazel E. Freeks FE, Kruger A. The Effect of the Lifeplan^®^ Programme on the Psychological Wellbeing of a Rural Community in South Africa, Journal of Psychology in Africa.2011;21:421–428.

[22] Rugira J, Nienaber AW, Wissing MP. Evaluating the effect of a psychosocial well-being programme for students at a Tanzanian university. African Journal for the Psychological Study of Social Issues. 2015;18:77–98

[23] Van Zyl LE, Rothmann S. Beyond smiling: the evaluation of a positive psychological intervention aimed at student happiness. Journal of Psychology in Africa. 2012;22:369–384.

[24] Brislin RW. Back translation for cross cultural research. Journal of cross cultural psychology. 1970;1:185–216.

[25] Van de Vijver FJR, Leung K. Methods and data analysis for cross-cultural research. 1997 Thousand Oaks, CA: Sage.

[26] Sousa VD, Rojjanasrirat W. Translation, adaptation and validation of instruments or scales for use in cross-cultural health care research: a clear and user-friendly guideline. Journal of Evaluation in Clinical Practice. 2011;17:268–274. 10.1111/j.1365-2753.2010.01434.x 20874835

[27] Muthén LK, Muthén BO. Mplus (Version 8.3). 1998–2018. Los Angeles, CA: 68. Muthén and 68. Muthén.

[28] Marsh HW, Morin AJS, Parker PD, Kaur G. Exploratory Structural Equation Modeling: An Integration of the Best Features of Exploratory and Confirmatory Factor Analysis. Annual Review of Clinical Psychology. 2014;10:85–110. 10.1146/annurev-clinpsy-032813-153700 24313568

[29] Morin AJS, Arens AK, Marsh HW. A Bifactor Exploratory Structural Equation Modeling Framework for the Identification of Distinct Sources of Construct-relevant Psychometric Multidimensionality. Structural Equation Modeling. 2016; 23:116–139.

[30] Joshanloo M, Jovanović V. The factor structure of the mental health continuum-short form (MHC-SF) in Serbia: An evaluation using exploratory structural equation modeling. Journal of Mental Health. 2017; 26:510–515. 10.1080/09638237.2016.1222058 27690711

[31] Perry JL, Nicholls AR, Clough PJ, Crust L. Assessing model fit: caveats and recommendations for confirmatory factor analysis and exploratory structural equation modeling. Measurement in Physical Education and Exercise Science. 2015;19:12–21. 10.1080/1091367x.2014.952370

[32] Reise SP. Invited Paper: The rediscovery of bifactor measurement models. Multivariate Behavioral Research. 2012; 47:667–96. 10.1080/00273171.2012.715555 24049214 PMC3773879

[33] Asparouhov T, Muthen B. Exploratory structural equation modeling. Structural equation modeling: a multidisciplinary journal. 2009;16:397–438. 10.1080/10705510903203516 PMC2921717 20717486

[34] Reise SP, Scheines R, Widaman KF, Haviland MG. Multidimensionality and structural coefficient bias in structural equation modeling: a bi-factor perspective. Educational and Psychological Measurement. 2013b;73:5–26. 10.1177/0013164412449831.

[35] Schutte L, Wissing MP. Clarifying the factor structure of the mental health continuum short form in three languages: A bifactor exploratory structural equation modeling approach. Society and Mental Health. 2017; 1–17. 10.1177/2156869317707793.

[36] Joshanloo M. A new look at the factor structure of the MHC-SF in Iran and the United States using exploratory structural equation modeling. Journal of Clinical Psychology. 2016a;72:701–713. 26990960 10.1002/jclp.22287

[37] Sass DA, Schmitt TA, Marsh HW. Evaluating model fit with ordered categorical data within a measurement invariance framework: A comparison of estimators. Structural Equation Modeling. 2014;21:167–180. 10.1080/10705511.2014.882658

[38] Van de Schoot R, Lugtig P, Hox J. A checklist for testing measurement invariance. European Journal of Developmental Psychology. 2012;9:486–492. 10.1080/17405629.2012.686740

[39] Byrne BM. Structural Equation Modeling with Mplus: Basic Concepts, Applications, and Programming. 2012 New York, NY: Routledge

[40] DiStefano C, Liu J, Jiang N, Shi D. Examination of the weighted root mean square residual: Evidence for trustworthiness? Structural Equation Modeling: A Multidisciplinary Journal. 2018;25:453–466. 10.1080/10705511.2017.1390394

[41] Hair J, Black WC, Babin BJ, Anderson RE. Multivariate data analysis. Upper saddle River, New Jersey: Pearson Education International 2010

[42] McDonald RP. The Theoretical Foundations of Principal Factor Analysis, Canonical Factor Analysis, and Alpha Factor Analysis. British Journal of Mathematical and Statistical Psychology. 1970;23:1–21.

[43] Sánchez-Oliva D, Morin AJS, Teixeira PJ, Carraça EV, Palmeira AL, Silva MN. A bifactor exploratory structural equation modeling representation of the structure of the basic psychological needs at work scale. Journal of Vocational Behavior. 2017;98:173–187.

[44] Nunnally JC. Psychometric Theory. 1978 New York, NY: McGraw-Hill.

[45] Perreira TA, Morin AJS, Hebert M, Gillet N, Houle SA., Berta W. The short form of the Workplace Affective Commitment Multidimensional Questionnaire (WACMQ-S): A bifactor-ESEM approach among healthcare professionals. Journal of Vocational Behavior. 2018;106:62–83. 10.1016/j.jvb.2017.12.004

[46] Kammann NR, Flett R. Affectometer 2: A scale to measure current level of general happiness. Australian Journal of Psychology. 1983;35:259–265.

[47] Wissing JAB, Wissing MP, Du Toit MM, Temane QM. Psychometric properties of various scales measuring psychological well-being in a South African context: The FORT 1 Project. Journal of Psychology in Africa. 2008;18:511–520.

[48] Tennant R, Joseph S, Stewart-Brown S. The Afectometer 2: a measure of positive mental health in UK populations. Quality of Life Research. 2007;6:687. 10.1007/s11136-006-9145-5 17268934

[49] Wissing MP, Thekiso SM, Stapelberg R, Van Quickelberge L, Choabi P, Moroeng C, et al Validation of three Setswana measures for psychological wellbeing. SA Journal of Industrial Psychology. 2010;36(2).

[50] Ingram RE, Wisnicki KS. Assessment of automatic positive cognition. Journal of Consulting and Clinical Psychology. 1988;56:898–902. 10.1037//0022-006x.56.6.898 3204200

[51] Jolly JB, Wiesner DC. Psychometric properties of the Automatic Thoughts Questionnaire-Positive with impatients adolescents. Cognitive Therapy and Research. 1996;20:481–498.

[52] Boelen PA. Psychometric Properties of the Dutch version of the Automatic Thoughts Questionnaire–Positive (ATQ–P). Cognitive Behaviour Therapy. 2007;36:23–33. 10.1080/16506070600874315 17364649

[53] Bryant FB, Baxter WJ. The structure of positive and negative automatic cognition. Cognition & Emotion. 1997;11(3), 225–258.

[54] Schwarzer R, Jerusalem M. Generalized Self-Efficacy scale In Weinman J., Wright S., & Johnston M., Measures in health psychology: A user’s portfolio. Causal and control beliefs. 1995;35–37. Windsor, UK: NFER-NELSON.

[55] Urban B. Entrepreneurial self-efficacy in a multicultural society: measures and ethnic differences. South African Journal of Industrial Psychology. 2006;32:2–10.

[56] Löve J, Moore CD, Hensing G. Validation of the Swedish translation of the general self-efficacy scale. Quality of Life Research. 2012;21:1249–1253. 10.1007/s11136-011-0030-5 21984467

[57] De las Cuevas C, Peñate W. Validation of the General Self-Efficacy Scale in psychiatric outpatient care. Psicothema. 2015; 27:410–415. 10.7334/psicothema2015.56 26493581

[58] Wu C. Factor analysis of the general self-efficacy scale and its relationship individualism /collectivism among twenty-five countries: Application of multi-level confirmatory factor analysis. Personality and Individual Differences. 2009;49:699–703.

[59] Zhou M. A revisit of general self-efficacy scale: uni- or multi-dimensional? Current Psychology. 2016, 35: 427–436.

[60] Kroenke K, Spitzer RL, Williams JBW. The PHQ-9: Validity of a Brief Depression Severity Measure. Journal of General Internal Medicine. 2001;16:606–613. 10.1046/j.1525-1497.2001.016009606.x 11556941 PMC1495268

[61] Adewuya AO, Ola BA, Afolabi OO. Validity of the patient health questionnaire (PHQ-9) as a screening tool for depression amongst Nigerian university students. Journal of Affective Disorders. 2006;96:89–93. 10.1016/j.jad.2006.05.021 16857265

[62] Granillo MT. Structure and function of the patient health questionnaire-9 among Latina and Non-Latina White female college students. Journal of the Society for Social Work and Research. 2012;3:80–93. 10.5243/jsswr.2012.6

[63] Guo B, Kaylor-Hughes C, Garland A, Nixon N, Sweeney T, Simpson S, et al Factor structure and longitudinal measurement invariance of PHQ-9 for specialist mental health care patients with persistent major depressive disorder: Exploratory structural equation modelling. Journal of Affective Disorders. 2017;219:1–8. 10.1016/j.jad.2017.05.020 28501679 PMC6602881

[64] Krause JS, Saunders LL, Bombardier C, Kalpakjian C. Comfirmatory factor analysis of Patient Health Questionnaire-9; a study of the participants from spinal cord injury models. PM&R. 2011;3:533–540. 21665166 10.1016/j.pmrj.2011.03.003

[65] Petersen JJ, Paulitsch MA, Hartig J, Mergenthal K, Gerlach FM, Gensichen J. Factor structure and measurement invariance of the Patient Health Questionnaire-9 for female and male primary care patients with major depression in Germany. J. Affect. Disord. 2015;170:138–142. 10.1016/j.jad.2014.08.053 25240840

[66] Subotić S, Knežević I, Dimitrijević S, Miholjčić D, Šmit S, Karać M, et al The factor structure of the Patient Health Questionnaire (PHQ-9) in a non-clinical sample. 2015. In S. Subotić (Ed.), STED 2015 Conference Proceedings–Psychology Section (pp. 20–28). Banja Luka, B&H: University for Business Engineering and Management.

[67] Familiar I, Ortiz-Panozo E, Hall B, Vieitez I, Romieu I, Lopez-Ridaura R, et al Factor structure of the Spanish version of the Patient Health Questionnaire-9 in Mexican women. International Journal of Methods in Psychiatric Research. 2015;24:74–82. 10.1002/mpr.1461 25524806 PMC6878506

[68] Merz EL, Malcame VL, Roesch SC, Riley N, Sadler GR. A multigroup confirmatory factor analysis of the Patient Health Questionnaire-9 among English- and Spanish-speaking Latinas. Cultural Diversity and Ethnic Minority Psychology. 2011;17:309–316. 10.1037/a0023883 21787063 PMC4210271

[69] Jung N, Wranke C, Hamburger K, Knauff M. How emotions affect logical reasoning: evidence from experiments with mood-manipulated participants, spider phobics, and people with exam anxiety. Front. Psychol. 2014;5:570 10.3389/fpsyg.2014.00570 24959160 PMC4050437

[70] Adewuya AO, Ola BA, Afolabi OO. Validity of the patient health questionnaire (PHQ-9) as a screening tool for depression amongst Nigerian university students. Journal of Affective Disorders. 2006;96:89–93. 10.1016/j.jad.2006.05.021 16857265

[71] Keum BT, Miller MJ, Inkelas KK. Testing the factor structure and measurement invariance of the PHQ-9 across racially diverse U.S. college students. Psychological Assessment. 2018;30:1096–1106. 10.1037/pas0000550 29565614

[72] Anum A, Adjorlolo S, Kugbey N. Depressive symptomatology in adolescents in Ghana: Examination of psychometric properties of the Patient Health Questionnaire-9. Journal of Affective Disorders. 2019;256:213–218. 10.1016/j.jad.2019.06.007 31181377

[73] World Health Organization. Integrating mental health into primary care A global perspective. Geneva: World Health Organization and World Organization of Family Doctors 2008a.

[74] Diener E, Emmons RA., Larsen RJ, Griffin S. The Satisfaction with Life Scale. Journal of Personality Assessment. 1985;49:71–75. 10.1207/s15327752jpa4901_13 16367493

[75] Wissing MP, van Eeden C. Empirical clarification of the nature of psychological well-being. South African Journal of Psychology. 2002; 32:32–44. 10.1177/008124630203200105

[76] Di Fabio AD, Gori A. Measuring Adolescent Life Satisfaction: Psychometric Properties of the Satisfaction with Life Scale in a Sample of Italian Adolescents and Young Adults. Journal of Psychoeducational Assessment. 2016;34:501–506.

[77] Schutte L, Negri, Delle Fave A, Wissing MP. Rasch analysis of the Satisfaction with Life Scale across countries: Findings from South Africa and Italy. Current Psychology. 2019. 10.1007/s12144-019-00424-5

