# Supplementary material for: Factorial validity of the Twi versions of five measures of mental health and well-being in Ghana
Source: PLoS One. 2020 Aug 11;15(8):e0236707. doi: 10.1371/journal.pone.0236707 (PMC7418998; doi:10.1371/journal.pone.0236707)
Supplement: S1 File — (DOC) [file pone.0236707.s001.doc]

***Affectometer 2 (****Kammann & Flett, 1983)*

**Hwɛ ɔkasamu a edidi so yi na yi nea ɛkyerɛ sɛnea w’atinka teɛ wɔ nnawɔtwe mmienu a atwam no mu**

| **ƆKASAMU** | | **Ɛnte saa koraa** | **Berɛ-ano berɛ-ano** | **Ɛtɔ da bi a** | **Mprɛ pii** | **Bere nyinaa** |
| --- | --- | --- | --- | --- | --- | --- |
| 1 | M’abrabɔ nam ɔkwan a ɛteɛ so | 0 | 1 | 2 | 3 | 4 |
| 2 | Obiara agya me hɔ wɔ mmerɛ a mempɛ sɛ meyɛ ankonam | 0 | 1 | 2 | 3 | 4 |
| 3 | Me wɔ gyedie sɛ metumi ayɛ deɛ mepɛ sɛ meyɛ biara | 0 | 1 | 2 | 3 | 4 |
| 4 | Me dwen tɔtee na me wɔ adeyɛ ho nyansa | 0 | 1 | 2 | 3 | 4 |
| 5 | Te sɛ obi a wadi nkuguo | 0 | 1 | 2 | 3 | 4 |
| 6 | Biribiara nyɛ anika biom | 0 | 1 | 2 | 3 | 4 |
| 7 | Me pɛ m’ankasa me ho asɛm | 0 | 1 | 2 | 3 | 4 |
| 8 | Biribiara ntumi nhaw me sɛ menyɛ biribi | 0 | 1 | 2 | 3 | 4 |
| 9 | Me ne nnipa a wɔbɛn me ntam yɛ | 0 | 1 | 2 | 3 | 4 |
| 10 | Ayɛ sɛ mfie papa a ɛwɔ m’abrabɔ mu atwam | 0 | 1 | 2 | 3 | 4 |
| 11 | Me daakye yɛ fɛ | 0 | 1 | 2 | 3 | 4 |
| 12 | M’ahwere afoforɔ ho anigye na wɔnfa me ho | 0 | 1 | 2 | 3 | 4 |
| 13 | Me wɔ ahoɔden ma ebi ka | 0 | 1 | 2 | 3 | 4 |
| 14 | Me nyaeɛ a anka mɛpɛ sɛ mɛsesa m’abrabɔ no fa bi | 0 | 1 | 2 | 3 | 4 |
| 15 | Me pɛ nwenwene ɛne sereɛ paa ara | 0 | 1 | 2 | 3 | 4 |
| 16 | M’adwen kyinkyin wɔ ɔkwan a ɛnhia so | 0 | 1 | 2 | 3 | 4 |
| 17 | Mɛ tumi asɔ ɔhaw biara a ɛbɛba ano | 0 | 1 | 2 | 3 | 4 |
| 18 | Ayɛ sɛ me bra apem anaa ɛnnya nkɔsoɔ | 0 | 1 | 2 | 3 | 4 |
| 19 | Me hu sɛ wɔdɔ me na wɔsan gye me di | 0 | 1 | 2 | 3 | 4 |
| 20 | Ɛyɛ me sɛ biribi ha me | 0 | 1 | 2 | 3 | 4 |

***SWLS (****Diener et al., 1985)*

**Kyerɛ sɛ wone ɔkasamu nnum yi yɛ adwene anaa nyɛ adwene. Yi akontahyɛde a egyina hɔ ma sɛ nea wone ɔkasamu no yɛ adwene fa (1 - wone nsɛm yi nnyɛ adwene koraa; 7 - wone nsɛm yi yɛ adwene paa)**

| Akwan hodoɔ biara so no, m’abrabɔ kɔ sɛdeɛ ɛsɛ sɛ ɛkɔ no ara. | 1 2 3 4 5 6 7 |
| --- | --- |
| M’abrabɔ mu nhyehyeɛ di mu. | 1 2 3 4 5 6 7 |
| M’ani sɔ sɛdeɛ m’abrabɔ te no. | 1 2 3 4 5 6 7 |
| Ɛde bedu saa bere yi, deɛ edi mu a mehia wɔ abrabɔ mu no, mensa aka. | 1 2 3 4 5 6 7 |
| Sɛ yɛma kwan sɛ memmɔ m’abrabɔ no biom a, mensesa hwee. | 1 2 3 4 5 6 7 |

***ATQ-P (****Ingram & Wisnicki, 1988)*

**Wɔakyerɛw ɔkasamu bi afa nsusuwii a eba nnipa adwene mu. Mepa wo kyɛw kenkan nsusuwii yi na kyere mprɛ dodoɔ, sɛ esi mpo a, a nsusuwii yi taa ba w’adwene mu wɔ nnawɔtwe baako a atwam no mu. Mepa wo kyɛw kenkan nsemfua no yiye na yi akontahyɛde a egyina hɔ ma nsusuwii ko no wɔ nhwɛsoɔ yi so:**

**1 = Ɛnte saa koraa**

**2 = Ɛtɔ mmere bi a**

**3 = Kakraa bi**

**4 = Mpɛn pii**

**5 = Bere nyinaa**

| **ƆKASAMU** | **1 = Ɛnte saa koraa** | **2 = Ɛtɔ mmere bi a** | **3 =**  **Kakraa bi** | **4 = Mpɛn pii** | **5 = Bere nyinaa** |
| --- | --- | --- | --- | --- | --- |
| 1.     M’atipɛnfoɔ bu me. | 1 | 2 | 3 | 4 | 5 |
| 2.     M’ani gye aseresɛm ho. | 1 | 2 | 3 | 4 | 5 |
| 3.     Me daakye besi gyinae pa. | 1 | 2 | 3 | 4 | 5 |
| 4.     Ɛbɛ si me yiye. | 1 | 2 | 3 | 4 | 5 |
| 5.     Me ho yɛ anika. | 1 | 2 | 3 | 4 | 5 |
| 6.     Me wɔ atinka pa mu. | 1 | 2 | 3 | 4 | 5 |
| 7.     Nnipa pii wɔ hɔ a wɔdwen me ho. | 1 | 2 | 3 | 4 | 5 |
| 8.     M’ani agye deɛ matumi ayɛ no ho. | 1 | 2 | 3 | 4 | 5 |
| 9.     Deɛ mɛ hyɛ aseɛ biara no mɛ wie. | 1 | 2 | 3 | 4 | 5 |
| 10. Nnoɔma pa pii wɔ me mu. | 1 | 2 | 3 | 4 | 5 |
| 11. M’ani gye abrabɔ ho. | 1 | 2 | 3 | 4 | 5 |
| 12. Me ne afoforɔ ntam yɛ. | 1 | 2 | 3 | 4 | 5 |
| 13. Me yɛ onipa a me tiri yɛ. | 1 | 2 | 3 | 4 | 5 |
| 14. Me wɔ namfoɔ a wɔboa me. | 1 | 2 | 3 | 4 | 5 |
| 15. Abrabɔ yɛ ahomeka. | 1 | 2 | 3 | 4 | 5 |
| 16. M’ani gye mpoatwa ho. | 1 | 2 | 3 | 4 | 5 |
| 17. Me ne afoforo abusuabɔ yɛ paa. | 1 | 2 | 3 | 4 | 5 |
| 18. Biribiara nni hɔ a ɛsɛ sɛ wo dwene ha wo ho. | 1 | 2 | 3 | 4 | 5 |
| 19. Me dwodwo me ho papaapa. | 1 | 2 | 3 | 4 | 5 |
| 20. M’abrabɔ da tɔɔtee. | 1 | 2 | 3 | 4 | 5 |
| 21. M'anigye sɛnea me teɛ ho. | 1 | 2 | 3 | 4 | 5 |
| 22. Me hwɛ me ho so yiye. | 1 | 2 | 3 | 4 | 5 |
| 23. Me fata sɛ mɛnya abrabɔ mu nnoɔma pa. | 1 | 2 | 3 | 4 | 5 |
| 24. Nna bɔne ntaa nsi. | 1 | 2 | 3 | 4 | 5 |
| 25. Nnoɔma papa bebree wɔ me mu a ɛso wɔ mfaso. | 1 | 2 | 3 | 4 | 5 |
| 26. Amaneɛ biara nni hɔ a ɛnni anidasoɔ. | 1 | 2 | 3 | 4 | 5 |
| 27. Memma ma ba mu mmu. | 1 | 2 | 3 | 4 | 5 |
| 28. Mede akokoduro kyerɛ m’adwene. | 1 | 2 | 3 | 4 | 5 |
| 29. M’abrabɔ kɔ so ara tu mpɔn. | 1 | 2 | 3 | 4 | 5 |
| 30. Nnɛ, matumi ayɛ nnoɔma pii. | 1 | 2 | 3 | 4 | 5 |

***GSEs (Schwarzer & Jerusalem, 1995****)*

**Nea edidi so yi yɛ ɔkasamu du fa wo ho a ɛyɛ nokware anaa ɛnyɛ nokware. Fa akontahyɛde hwɛsoɔ yi (1-4) na kyerɛ sɛ neɛ wone ɔkasamu no yɛ adwene fa. Mepa wo kyɛw fa nokware die ma muae.**

|  | **ƆKASAMU** | **Ɛnyɛ nokware koraa** | **Ɛntaa nyɛ nokware** | **Ɛyɛ nokware kakra** | **Ɛyɛ nokware paa** |
| --- | --- | --- | --- | --- | --- |
| 1. | Sɛ me bɔ meho mmɔden paa ara a, mɛtumi asɔ ɔhaw denden biara ano. | 0 | 1 | 2 | 3 |
| 2. | Sɛ obi ko tia me a, mɛtumi afa akwan biara so anya deɛ mepɛ. | 0 | 1 | 2 | 3 |
| 3. | Ɛyɛ mmrɛ ma me sɛ mɛdi me botaeɛ ahorow so na m’atumi ayɛ nea ɛda m’akoma so. | 0 | 1 | 2 | 3 |
| 4. | Mewɔ akokoɔduro sɛ, sɛ nsɛm bi to me mpofirim a, mɛtumi adi so yiye. | 0 | 1 | 2 | 3 |
| 5. | Ɛnam me nimdeɛ so nti, menim sɛdeɛ metumi ne mpofirim haw adi asie. | 0 | 1 | 2 | 3 |
| 6. | Mɛtumi asɔ ɔhaw pii ano sɛ mede ahoɔden pii kɔ so a. | 0 | 1 | 2 | 3 |
| 7. | Sɛ mewɔ ɔhaw bi mu a, memmɔ hu efiri sɛ mewɔ akadeɛ a metumi de agyina. | 0 | 1 | 2 | 3 |
| 8. | Sɛ ɔhaw ba me so a, me tumi nya nsoano pii. | 0 | 1 | 2 | 3 |
| 9. | Sɛ mewɔ ɔhaw mu a, mpɛn pii no medwen ɔkwan a mede besi ano. | 0 | 1 | 2 | 3 |
| 10. | Deɛ ɛbɛba m’akwan mu biara no, metumi siesie mpɛm pii. | 0 | 1 | 2 | 3 |

***PHQ-9 (Kroenke, Spitzer, & Williams, 2001)***

| **Wɔ nnawɔtwe mmienu a atwam no mu no, mmerɛ dodoɔ sɛn na ɔhaw a edidi so yi aba w’akwan mu?** | **Ɛnte saa koraa** | **Nna bebree** | **Ɛboro nna no mu fa** | **Ayɛ sɛ dabiara da** |
| --- | --- | --- | --- | --- |
| 1. Anigye a wode yɛ adeɛ wɔ fam paa ara | 0 | 1 | 2 | 3 |
| 2. W’ahokeka kɔ fam/wosusum boto/wonni anidasoɔ | 0 | 1 | 2 | 3 |
| 3. Wo brɛ ansa na wada anaa sɛ wo da boroso | 0 | 1 | 2 | 3 |
| 4. Wo te brɛ/w’ahoɔden so ate | 0 | 1 | 2 | 3 |
| 5. Wontumi nnidi anaa wo didi boroso | 0 | 1 | 2 | 3 |
| 6. W’ani nnye wo ho/sɛ wo yɛ nkugu di fo/ sɛ w’adi wo ho anaa w’abusua hwammɔ | 0 | 1 | 2 | 3 |
| 7. Ɛyɛ wo den sɛ wode w’adwene bɛsi biribiara so te sɛ krataa akenkan /TV hwɛ | 0 | 1 | 2 | 3 |
| 8. Wo nante anaa wo kasa bɔkɔɔ ma afoforɔ ani ba wo so anaa w’ahoyeraw ama wo nante pii | 0 | 1 | 2 | 3 |
| 9. Adwen bi sɛ anka wowu a, ɛyɛ / anaa woredi wo ho awu ɔkwan bi so | 0 | 1 | 2 | 3 |
